# *GIGYF1* loss of function is associated with clonal mosaicism and adverse metabolic health

**DOI:** 10.1038/s41467-021-24504-y

**Published:** 2021-07-07

**Authors:** Yajie Zhao, Stasa Stankovic, Mine Koprulu, Eleanor Wheeler, Felix R. Day, Hana Lango Allen, Nicola D. Kerrison, Maik Pietzner, Po-Ru Loh, Nicholas J. Wareham, Claudia Langenberg, Ken K. Ong, John R. B. Perry

**Affiliations:** 1 MRC Epidemiology Unit, Institute of Metabolic Science, University of Cambridge, Cambridge, UK; 2 Division of Genetics, Department of Medicine, Brigham and Women’s Hospital and Harvard Medical School, Boston, MA USA; 3 Broad Institute of MIT and Harvard, Cambridge, MA USA

**Keywords:** Genome-wide association studies, Genomic instability, Type 2 diabetes

## Abstract

Mosaic loss of chromosome Y (LOY) in leukocytes is the most common form of clonal mosaicism, caused by dysregulation in cell-cycle and DNA damage response pathways. Previous genetic studies have focussed on identifying common variants associated with LOY, which we now extend to rarer, protein-coding variation using exome sequences from 82,277 male UK Biobank participants. We find that loss of function of two genes—*CHEK2* and *GIGYF1*—reach exome-wide significance. Rare alleles in *GIGYF1* have not previously been implicated in any complex trait, but here loss-of-function carriers exhibit six-fold higher susceptibility to LOY (OR = 5.99 [3.04–11.81], p = 1.3 × 10^−10^). These same alleles are also associated with adverse metabolic health, including higher susceptibility to Type 2 Diabetes (OR = 6.10 [3.51–10.61], *p* = 1.8 × 10^−12^), 4 kg higher fat mass (*p* = 1.3 × 10^−4^), 2.32 nmol/L lower serum IGF1 levels (*p* = 1.5 × 10^−4^) and 4.5 kg lower handgrip strength (*p* = 4.7 × 10^−7^) consistent with proposed *GIGYF1* enhancement of insulin and IGF-1 receptor signalling. These associations are mirrored by a common variant nearby associated with the expression of *GIGYF1*. Our observations highlight a potential direct connection between clonal mosaicism and metabolic health.

## Abstract (web summary)

Mosaic loss of chromosome Y (LOY) is a common form of clonal mosaicism in leukocytes. Here, the authors extend genetic association analyses to rare variation using exome-sequence data from 82,277 males, finding that loss-of-function alleles in *GIGYF1* are associated with six-fold higher susceptibility to both LOY and Type 2 Diabetes.

## Introduction

Mosaic loss of the Y chromosome in leukocytes (LOY) is the most common form of clonal mosaicism, first noted over fifty years ago^1,2^. It has been associated with the risk of a number of complex diseases and traits, however, the biological mechanisms underpinning these observations are unclear^3–10^. Like other forms of clonal mosaicism, LOY is strongly associated with age, reflecting greater opportunity for mitotic errors in hemopoietic stem cell division and subsequent clonal expansion to occur. Predisposition to LOY also has a heritable component and to date, over 150 associated common genetic variants have been identified^11–14^. These loci have implicated genes involved in cell-cycle fidelity and DNA damage response (DDR), supporting the idea that LOY is a readily detectable manifestation of subtle defects in these processes^12,13^. We have hypothesized that the predisposition to genomic instability that is shared across multiple cell types, including leukocytes, may explain the observational associations between LOY and other health outcomes^13^. This concept is most apparent for *CHEK2* loss of function, which both promotes LOY in men and extends reproductive life in women through the shared mechanism of inhibiting DNA damage sensing and apoptosis. Identifying novel genetic determinants associated with LOY has the potential, therefore, to not only increase our knowledge of clonal hematopoiesis but also to identify loci that underlie susceptibility to other complex traits through shared biological mechanisms. We previously demonstrated this with Type 2 Diabetes (T2D), where overlap with LOY highlights loci which likely impact cellular homeostasis in metabolic tissues. For example, alleles in *CCND2* increase the risks of both T2D and LOY^13^, with this gene encoding the major D-type cyclin that is expressed in pancreatic β-cells and is essential for adult β cell growth^15^.

To date, genetic studies for LOY have focussed on genotype-array imputed common genetic variation, which largely misses the contributions of rarer, often more deleterious, alleles^11–13^. Here, we report an exome-sequence GWAS for LOY, assessing the role of rare protein-coding variation. We extend and confirm previous observations supporting the role of *CHEK2* and additionally identify an association with *GIGYF1* loss of function, highlighting an intriguing link between LOY and metabolic health.

## Results

Previous studies have quantified LOY using either a quantitative measure derived from the mean log2-transformed R ratio of signal intensity (mLRR-Y)^11^ or more recently a more-powered dichotomous measure (PAR-LOY) using allele-specific genotyping intensities in the sex chromosome pseudo-autosomal region (PAR)^13^. We note that both measures are proxies for the abundance of Y chromosome genetic material in the measured biological samples, derived from intensity data which contains much experimental ‘noise’. As these measures are independent—one relies on PAR genotypes only whilst the other excludes them—we hypothesized that an aggregate of the two would further help improve the signal-to-noise ratio of these measures and therefore increase statistical power to detect genetic associations. We name this combined quantitative measure PAR-LOYq (Online Methods) and estimated it in the same UK Biobank participants who were previously studied for PAR-LOY (*N* = 205,011 men). As expected PAR-LOYq calls provided a more powerful measure for discovery analysis, with a median 10.6% increase in chi-square association statistic for the 156 LOY loci previously identified by PAR-LOY (Supplementary Data 1)^13^.

To identify genes associated with LOY, we performed gene burden analyses for PAR-LOYq in 82,277 male UK Biobank participants with exome sequence data (Online Methods). Two models were tested exome-wide, by collapsing together rare (MAF < 0.5%) loss of function or moderate-impact variants in each individual gene. The association of the burden test in two genes, *CHEK2* and *GIGYF1*, were statistically significant exome-wide (*p* < 1.6 × 10^−6^) across these analyses (Supplementary Data 2, Fig. 1). Loss of function variants in *CHEK2* (*N* = 543 carriers, effect = 0.23 SD higher PAR-LOYq between rare allele carriers vs. non-carriers, *p* = 3.4 × 10^−9^) have previously been implicated with LOY as the most common frameshift variant (1100delC, MAF~0.2%) is well captured by GWAS imputation and directly genotyped on the UKBB array. This single variant accounted for 76% of loss of function carriers and the *CHEK2* association was nominally significant when it was excluded (*p* = 0.02, effect = 0.18 SD). An independent burden test of rare moderate-impact alleles in *CHEK2* (not including 1100delC and other loss of function alleles) was also associated with PAR-LOYq (Supplementary Data 2, *N* = 1057 carriers, effect = 0.11 SDs, *p* = 1.7 × 10^−4^).

**Fig. 1:**
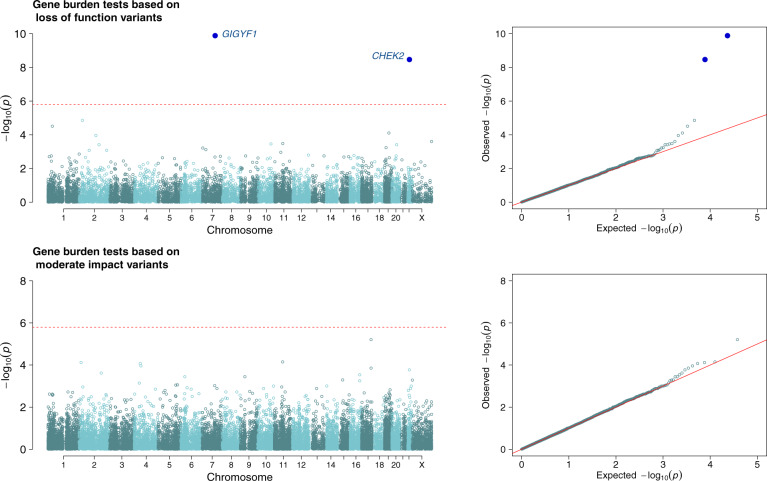
Manhattan and Quantile-Quantile (QQ) plots for exome-wide gene burden test statistics. The dashed red line denotes the exome-wide significance threshold (*p* < 1.6 × 10^−6^). Burden tests performed in *N* = 82,277 males.

*GIGYF1* loss of function (*N* = 40 male carriers) was associated with a 0.93 SD (0.64–1.21, *p* = 1.3 × 10^−10^) higher PAR-LOYq. This burden signal combined the effects of 27 rare variants (Supplementary Data 3); a single base insertion frameshift with 10 carriers, 4 doubletons, and 22 singleton rare alleles. No individual variant was more significant than the overall *GIGYF1* test result, which remained significant in a leave-one-out analysis of each variant (Supplementary Data 4). Rare moderate-impact alleles were not associated with LOY in aggregate (*p* = 0.70), however, several individual moderate-impact variants exhibited nominally significant associations (Supplementary Data 3). We note that missense alleles likely represent a heterogeneous collection of loss of function, gain of function, and benign effects. As with *CHEK2*, bioinformatic filters poorly predicted which missense variants in *GIGYF1* were associated with LOY (Fig. S1). Further genome-wide burden analyses in STAAR (see methods), weighting each variant by its CADD score, did not identify additional LOY-associated genes (Supplementary Data 5).

We next performed several sensitivity analyses to further explore the genetic architecture of this *GIGYF1-*LOY association. Firstly, we observed consistent effects using the two previous LOY traits, with a 6-fold (OR = 5.99 [3.04–11.81], *p* = 6 × 10^−7^) higher risk of a PAR-LOY dichotomous call and a −0.038 (~0.81 SD, *p* = 8.8 × 10^−9^) reduction in mLRRy. Secondly, in a sensitivity analysis, PAR-LOYq association results were highly consistent when excluding multi-allelic sites (*p* = 8.4 × 10^−9^) or indels (*p* = 9.9 × 10^−3^) and when restricting to high-confidence loss of function variants defined by LOFTEE (*p* = 4.1 × 10^−13^)^16^. Sequencing quality control parameters for each individual variant appeared robust (Supplementary Data 3). Thirdly, we reproduced the same association signal using a second analytical pipeline implemented in STAAR (*p* = 1.73 × 10^−10^)^17^. Finally, we showed that *GIGYF1* loss of function was not associated with any genetically derived principal component and carriers were geographically dispersed across the UK (Figs. S2–S3).

*GIGYF1* is named after its known binding to growth factor receptor-bound protein 10 (GRB10) and interacts with both the insulin and *IGF1* receptors^18^. We, therefore, postulated that loss of function alleles may also impact metabolic health, and, therefore, tested *GIGYF1* burden test association analyses across 17 metabolic-health related traits in men and women (Supplementary Data 6). *GIGYF1* loss of function (*N* = 64 carriers) was associated with higher susceptibility to T2D (OR = 6.10 [3.51–10.61], p = 1.8 × 10^−12^) and higher acute and longer-term average levels of glycemia in non-diabetic individuals (random glucose *p* = 2.6 × 10^−5^ and HbA1c *p* = 6.6 × 10^−7^). Of the 64 carriers, 19 (30%) had T2D, compared to 7.1% in the population of UK Biobank in whom sequence data was available. Carrier status was also associated with a 1.85 kg/m^2^ higher body mass index (*p* = 5.3 × 10^−4^), 4 kg higher fat mass (*p* = 1.3 × 10^−4^), 1.85 kg higher lean mass (*p* = 5.2 × 10^−3^), 0.04 higher waist-to-hip ratio (*p* = 1.8 × 10^−6^), −0.01 lower sitting to standing height ratio (*p* = 4.3 × 10^−7^), 4.5 kg lower grip strength (*p* = 4.7 × 10^−7^) and 2.32 nmol/L lower serum IGF1 levels (*p* = 1.5 × 10^−4^). The T2D association was largely unattenuated by adjustment for BMI (OR 5.07 [2.78−9.27] *p* = 8.9 × 10^−11^) and the clinical characteristics of the rare allele carriers with T2D did not provide any evidence of a phenotype distinct from typical T2D (Supplementary Data 7). Notably, GIGYF1 loss of function was not associated with birthweight, puberty timing, childhood body size, or adult height (*p* > 0.05).

We next examined whether common genetic variation in *GIGYF1* was also associated with LOY and metabolic health parameters. We observed that an intergenic variant (rs221781, MAF = 11% Supplementary Data 8 and Fig. S4) ~25 kb from *GIGYF1* was significantly associated with higher glucose (*P* = 4.80 × 10^−15^) and HbA1c (*P* = 3.40 × 10^−10^) in UK Biobank. This same allele was associated with a higher risk of T2D^19^ (OR adj BMI = 1.06 (1.04–1.09), *p* = 8.50 × 10^−8^) and LOY (*p* = 3.00 × 10^−6^), but with lower circulating LDL (*p* = 3.40 × 10^−10^) and HDL (*p* = 1.90 × 10^−18^) levels. The variant was not associated with BMI (*p* = 0.09). The lead signal for T2D (rs221781) is also the lead conditionally independent eQTL for *GIGYF1* across a number of GTEx tissues including subcutaneous adipose (Fig. S5), in which we observed that higher expression of *GIGYF1* was associated with a lower risk of T2D. The lead eQTL for *GIGYF1* is rs221792 in cultured fibroblasts (*p* = 1.3 × 10^−32^) which is in high LD (*r*^2^ = 0.71, *D*’ = 1) with rs221781. The association of common *GIGYF1* variants with T2D was also confirmed in Million Veteran Program data, in which we found a previously reported lead SNP for T2D was in high LD with rs221781 (rs534043, *r*^2^ = 1, *P* = 8.03 × 10^−10^) with a consistent direction of effect^20^.

## Discussion

In summary, this exome-wide approach identified rare loss of function alleles in *GIGYF1* exhibiting an effect on LOY ~5 times larger than any genetic variants previously identified by GWAS. Similarly, these alleles confer effect sizes on a number of metabolic outcomes far larger than those previously identified by imputed GWAS and other smaller sequencing studies. For example, rare variants in *PDX1*, *CCND2, SLC30A8*, and *PAM* are associated with double the odds of T2D^21–23^, whereas *GIGYF1* loss of function is associated with a six-fold increased risk (OR = 5.96 [3.43–10.38]). The majority of common variants associated with T2D confer much more modest effects (OR < 1.5)^19^.

*GIGYF1* encodes a member of the gyf family of adaptor proteins. It binds growth factor receptor-bound 10 (GRB10), which is another adaptor protein that binds activated insulin receptors and insulin-like growth factor-1 (IGF-1) receptors to negatively regulate receptor signaling, metabolic responses, and IGF1-induced mitogenesis^18,24,25^. Transfection of cells with GRB10-binding fragments of GIGYF1 leads to greater activation of both the insulin receptor and the IGF-1 receptor^26^. Our findings relating loss of function variants in *GIGYF1* to metabolic and anthropometric outcomes are broadly consistent with the notion that in individuals carrying two functional copies of this gene, *GIGYF1* enhances insulin and IGF-1 receptor signaling, leading to greater handgrip strength (relative to loss of function carriers), sitting height and circulating IGF-1 levels (due to increased insulin signaling), and lower % body fat, WHR, HbA1c, glucose levels, and susceptibility to T2D. We previously highlighted the potential role of IGF signaling in promoting chromosomal instability and the cellular accumulation of DNA damage and reported that genetically higher IGF-1 levels are related to greater LOY^27^. It may therefore appear paradoxical that here we find that loss of function in *GIGYF1* (putatively leading to decreased IGF-1 signaling) should be associated with increased rather than decreased LOY. We hypothesize that *GIGYF1* might enhance DDR mechanisms to protect DNA integrity in the face of IGF-1-mediated tissue growth and differentiation. *GIGYF1* and the related protein *GIGYF2* are implicated in translational repression^28^ and translation-coupled mRNA decay^29^, which suggests that they may have biological roles beyond insulin and IGF-1 receptor signaling. Although *GIGYF1* is broadly expressed^30^, the lack of associations in our data with some established IGF-1-related traits, such as birth weight and adult height, might reflect tissue or developmental specificity in its effects. We anticipate that future experimental work will shed light on these questions to better understand the links between clonal mosaicism and metabolic health.

## Methods

### Phenotype definitions

Until now, there were two established mLOY estimation methods based on SNP-array data: (1) the median or mean of log R ratio (mLRR-Y) genotyping intensity values of the probes on the male-specific regions of chromosome Y (MSY); and (2) the phase-based computational method that estimates allelic imbalance using only the pseudoautosomal regions (PAR-LOY) detailed previously^13^. The mLRR-Y and PAR-LOY are independent approaches as they are estimated from non-overlapping regions of the Y chromosome. Although there is a considerable correlation in the LOY estimates produced by these two methods, we sought to combine the independent information considered by the two approaches to increase power for genetic association analyses. We combined PAR-LOY and mLRR-Y with an additional measure, the estimated fraction of cells with LOY(AF-LOY) which was estimated when generating PAR-LOY^13^. Our new combined call of LOY (PAR-LOYq) is defined as PAR-LOY + (3*AF-LOY) – (3*mLRR-Y) (cropped to the range [0,2]). The intuition behind this formula is to augment the binary PAR-LOY variable by up-weighting individuals who have a larger LOY cell fraction (as estimated by AF-LOY and mLRR-Y), which may be more strongly associated with risk alleles.

We compared the performance of the three LOY estimates derived from the genotyping array data using the full set of male UKBB participants^13^. All UK Biobank participants provided written informed consent, the study was approved by the National Research Ethics Service Committee North West—Haydock, and all study procedures were performed in accordance with the ethical principles for medical research from the World Medical Association Declaration of Helsinki. We performed association testing with age and ever smoking status, which are two established risk factors for LOY^31,32^, and the 156 previously reported LOY-associated loci^13^. For both age and smoking status, PAR-LOYq outperformed the two established mLOY estimation methods using the same sample; the *t*-test statistic of PAR-LOYq for age increased by 65.4% and 5.2%, respectively, and the t-test statistic of PAR-LOYq for ever smoking status increased by 44.9% and 11.1%, respectively. Improvement of PAR-LOYq over PAR-LOY was also evaluated for the 156 previously identified variants by assessing the median improvement in chi-square statistic.

Participants were classified as cases of Type 2 diabetes (T2D) according to the previously published UKBB probable T2D algorithm^33^ based on baseline self-reported diabetes or medications, in addition to evidence from electronic health records (Hospital Episode Statistics or Death Registration) consistent with T2D (International Statistical Classification of Diseases and Related Health Problems Tenth Revision code E11). Any possible or probable Type 1 diabetes cases were excluded. Controls were participants without evidence of T2D as defined above. The GWAS on random glucose and HbA1C—using the BOLT-LMM pipeline described below—was performed after excluding individuals with our defined T2D criteria. The T2D test statistic for the common variant was taken from the DIAMANTE consortium GWAS meta-analysis^19^. All other phenotypes used in this study were available from UK Biobank and any applied transformations are described in the relevant results tables.

### UK Biobank exome-sequence data processing and QC

We downloaded VCF and PLINK format files for whole-exome sequencing (WES) data of 200,643 UK Biobank participants, which were made available in October 2020. The overview of this 200 K WES release is described at https://biobank.ndph.ox.ac.uk/ukb/label.cgi?id=170. Details of sequence data processing (read alignment, variant calling, etc.) are described in papers of Szustakowski et al. [10.1101/2020.11.02.20222232] and Yun et al. [10.1101/2020.02.10.942086]

We merged individual VCF files into a single VCF file of each chromosome using BCFtools v1.9^34^. We converted each chromosome file losslessly to a GDS (Genomic Data Structure) format file (an RData object) using the seqVCF2GDS() function from the R package SeqArray v1.30.0^35^. We used SeqArray package and GDS data object to extract the dosage matrix and perform additional variant and genotype level filtering below. Such genotype data processing is faster than using a flat text VCF file because GDS is implemented using an optimized C++ library and a high-level R interface is provided by the platform-independent R package gdsfmt^35,36^.

We used SeqArray package to calculate and extract the QC metrics. Firstly, we identified and flagged 7,913,671 on-target variants (those defined by the xgen_plus_spikein.GRCh38.bed file genomic coordinates) among the total of 15,916,398 called variants on autosomes and chromosome X. The UKBB released VCF file has a number of QC metrics that can be used for variant site and individual genotype filtering: QUAL (variant site-level quality score, Phred scale); AQ (variant site-level allele quality score reflecting evidence for each alternate allele, Phred scale); DP (individual genotype call-level approximate read depth (reads with MQ = 255 or with bad mates had already been filtered out)); AD (individual genotype call-level allelic depths for the ref and alt alleles in the order listed); GQ (individual genotype call-level Genotype Quality, Phred scale). We additionally calculated the site-level genotype missingness (the number of samples at each site without genotype call).

After generating the summary statistics of QUAL and AQ metrics, we noted that the released UKBB 200 K WES data already had some QC filters applied. The values of QUAL and AQ ranged from 20 (error rate = 1%) to 99 (error rate <0.0001%) with mean 44.5 (error rate <0.01%). For all chromosomes, the distributions of the values of QUAL and AQ are nearly the same. We decided not to apply additional stricter filters on these two site-level metrics. We calculated summary statistics (minimum, maximum, mean, and 1st, 2nd, and 3rd quartile) for DP and GQ for each variant based on all 200,643 samples for autosomes and 110,438 female samples for the X chromosome. We recorded the number of samples with GQ < 20 at each variant. We calculated allelic balance for each heterozygous genotype calls at on-target bi-allelic sites (ABratio), defined as the number of alternate allele’s reads (provided in the AD field) divided by the total depth which equals the sum of reading depths of reference allele and alternative allele. We then generated the same per-site summary statistics as above for ABratio. We defined and excluded a heterozygous genotype call as imbalanced if ABratio ≤ 0.25 or ABratio ≥ 0.8.

In our sensitivity analysis, we applied three variant-level filters to exclude variants at potentially poorly performing sites: filter 1: >5% missingness (samples without genotype calls); filter 2: the maximum of the read depth of genotype calls (DP) across samples <10; and filter 3: >20% genotype calls with GQ < 20. After applying these three filters, 1,161,679 (7.3%) of the initial 15,916,398 variants, and 96,640 (1.2%) of the 7,913,671 on-target variants were excluded. For the variants included in our variant-set analysis, we also generated the same QC metrics restricted only to rare allele carriers. Ultimately all of these metrics were used to filter out variants in sensitivity analyses that were initially performed using the default QC parameters applied to the UKBB released dataset.

### Variant annotation and definition of gene burden sets

We annotated variants released in UK Biobank (UKBB) 200 K whole-exome sequencing (WES) VCF files using the Ensembl Variant Effect Predictor tool release 99 based on build hg38^37^. For each uploaded variant, the default VEP features include consequence and impact of the variant, overlapping gene, position at cDNA and protein level, and amino acid change, if applicable. In addition to the default features, the following plugins from VEP were used: (i) SIFT^38^, which predicts whether an amino acid substitution affects protein function based on sequence homology and the physical properties of the amino acid, (ii) Polyphen-2^39^, which predicts possible impact of an amino acid substitution on the structure and function of a protein, (iii) CADD^40^ which provides deleteriousness prediction scores for all variants based on diverse genomic features, and (iv) LOFTEE^16^ which provides loss of function prediction for variants. The variants were annotated for every available overlapping transcript in Ensembl. We used the most severe variant definition for each variant-gene pair, which provides the annotation of the variant for the transcript it has the most severe consequence on.

We defined loss of function variants as those with ‘high impact’ prediction by VEP. This includes: frameshift variants, transcript ablating or transcript amplifying variants, splice acceptor or splice donor variants, stop lost, start gained, or stop gained variants. ‘Moderate impact’ variants include missense variants, inframe deletion or insertions, missense variants, and protein-altering variants.

### Gene association testing

Gene burden scores were created by collapsing all annotated rare alleles together to define a binary call denoting whether an individual carries none vs. one or more rare alleles at a given gene. Reported effect estimates therefore represent the trait difference between carriers and non-carriers of these alleles. These dummy variables were then transformed into BGEN file format genotype call files for association testing using a linear mixed model implemented in BOLT-LMM^41^ to account for cryptic population structure and relatedness. Only autosomal genetic variants that were common (minor allele frequency (MAF) >1%), passed quality control in all 106 batches, and were present on both genotyping arrays were included in the genetic relationship matrix (GRM). Genotyping chip, age at baseline, and ten genetically derived principal components were included as covariates. Samples were excluded from analysis if they failed UK Biobank quality control parameters, were of non-European ancestry or if the participant withdrew consent from the study.

### Secondary association testing

We applied STAAR (variant-Set Test for Association using Annotation infoRmation)^17^ as a secondary analytical approach for associated genes. STAAR is a general framework for performing a rare variants association study at scale, suitable for whole exome or genome population-level datasets such as UKBB. STAAR accounts for population structure and relatedness, by fitting linear and logistic mixed models for quantitative and dichotomous traits. It takes as input individual data frames for genotypes, phenotypes, covariates including age, age^2^, sex, chip, PC1-PC10 were generated from the SNP array data and (sparse) GRM.

We used the basic function of STAAR (with CADD-score weighting additionally performed in a sensitivity analysis) and set the thresholds of MAF ≤0.5% and ≥2 rare variants count in a gene. The output of STAAR provides *p*-values for a number of different rare variant set burden tests including SKAT(sequence kernel association test), Burden test, and ACAT-V(set-based aggregated Cauchy association test). In addition, STAAR provides an omnibus test result by using the combined Cauchy association test to aggregate the association across the different tests.

To ensure that the individual gene-level result is not disproportionally affected by a single variant of considerably larger effect and that the others are part of the same variant set, we performed a drop-one-out analysis using STAAR for our target gene.

Effect estimates for dichotomous traits were estimated by using logistic regression performed in R (3.3.3). Where these are reported they include the *p*-value obtained from the linear mixed-model generated by BOLT-LMM.

### Reporting summary

Further information on research design is available in the Nature Research Reporting Summary linked to this article.

## Supplementary information

Supplementary Information

Description of Additional Supplementary Files

Supplementary Data 1

Supplementary Data 2

Supplementary Data 3

Supplementary Data 4

Supplementary Data 5

Supplementary Data 6

Supplementary Data 7

Supplementary Data 8

Reporting Summary

## Data Availability

All individual-level data used in this study are available from the UK Biobank study upon application (www.ukbiobank.ac.uk). Full exome-wide summary statistics are reported in the supplement. The exome sequence data resource is described here: https://biobank.ndph.ox.ac.uk/ukb/label.cgi?id=170 and mosaic LOY calls here: https://biobank.ndph.ox.ac.uk/ukb/dset.cgi?id=3094. European GWAS meta-analysis summary results from the Million Veteran Program and other biobanks are available via dbGaP (NCBI dbGaP analysis accession pha004945, available to download from https://ftp.ncbi.nlm.nih.gov/dbgap/studies/phs001672/analyses/). DIAMANTE consortium GWAS meta-analysis results are available to download from the DIAGRAM consortium website (https://www.diagram-consortium.org/downloads.html).
