# Supplementary material for: GIGYF1 loss of function is associated with clonal mosaicism and adverse metabolic health
Source: Nat Commun. 2021 Jul 7;12:4178. doi: 10.1038/s41467-021-24504-y (PMC8263756; doi:10.1038/s41467-021-24504-y)

Supplementary Information for "*GIGYF1* loss of function is associated with clonal mosaicism and adverse metabolic health"

Yajie Zhao[1], Stasa Stankovic[1], Mine Koprulu[1], Eleanor Wheeler[1], Felix R Day[1], Hana Lango Allen[1], Nicola D Kerrison[1], Maik Pietzner[1], Po-Ru Loh[2,3], Nicholas J Wareham[1], Claudia Langenberg[1], Ken K Ong[1] and John R.B. Perry[1]

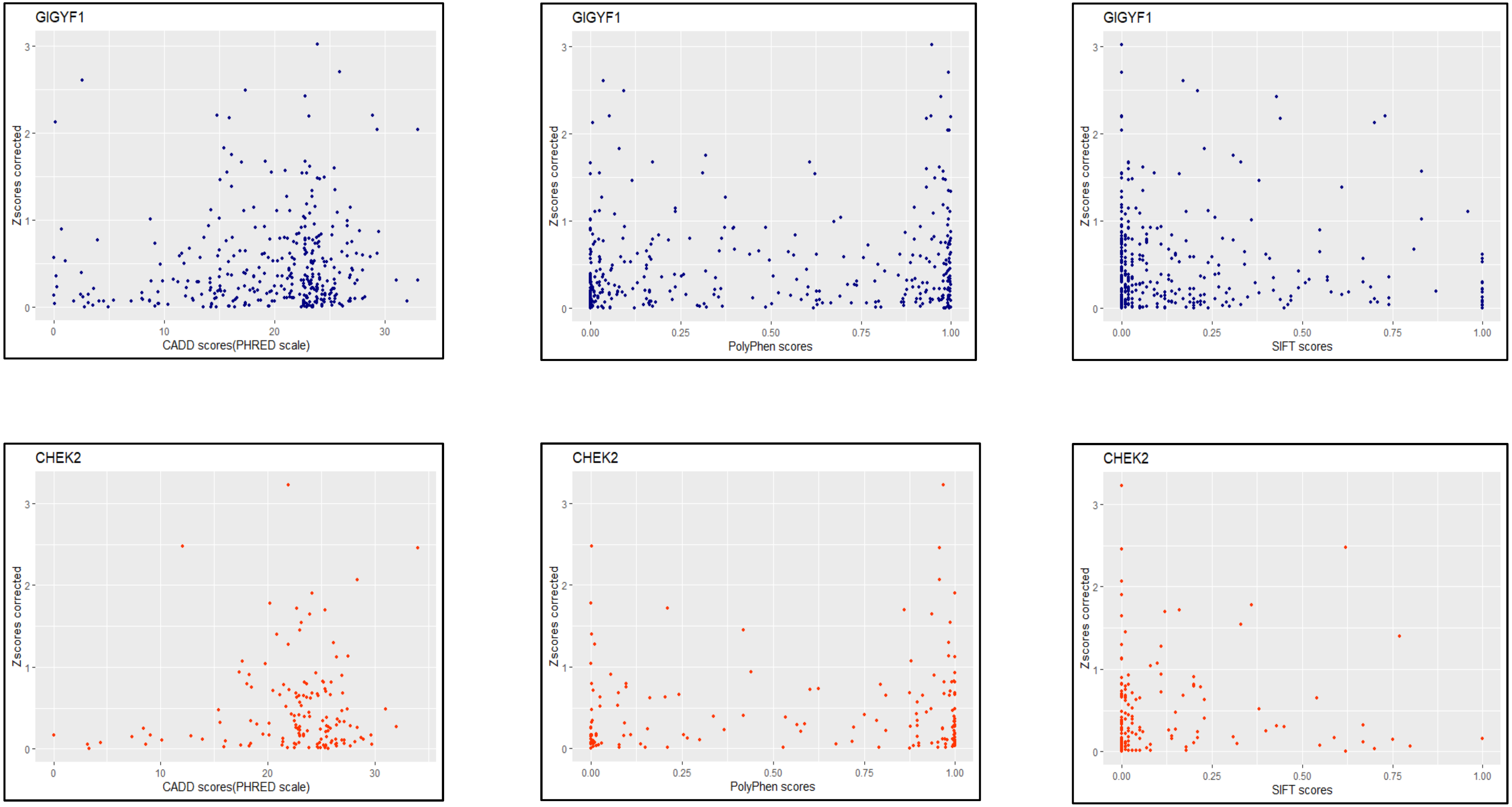

Figure S1 |Relationship between bioinformatically predicted function and LOY association for *GIGYF1* and *CHEK2* moderate-impact variants. Y-axis shows the PAR-LOYq association of each variant assessed by absolute Z-score divided by minor allele count.

Figure S2 |Impact of GIGYF1 loss of function carriers on genetically-defined principal components. GIGYF1 carriers are highlighted in red, all other analysed samples in black. Analysis performed in maximum available sample-size (N=184,972).

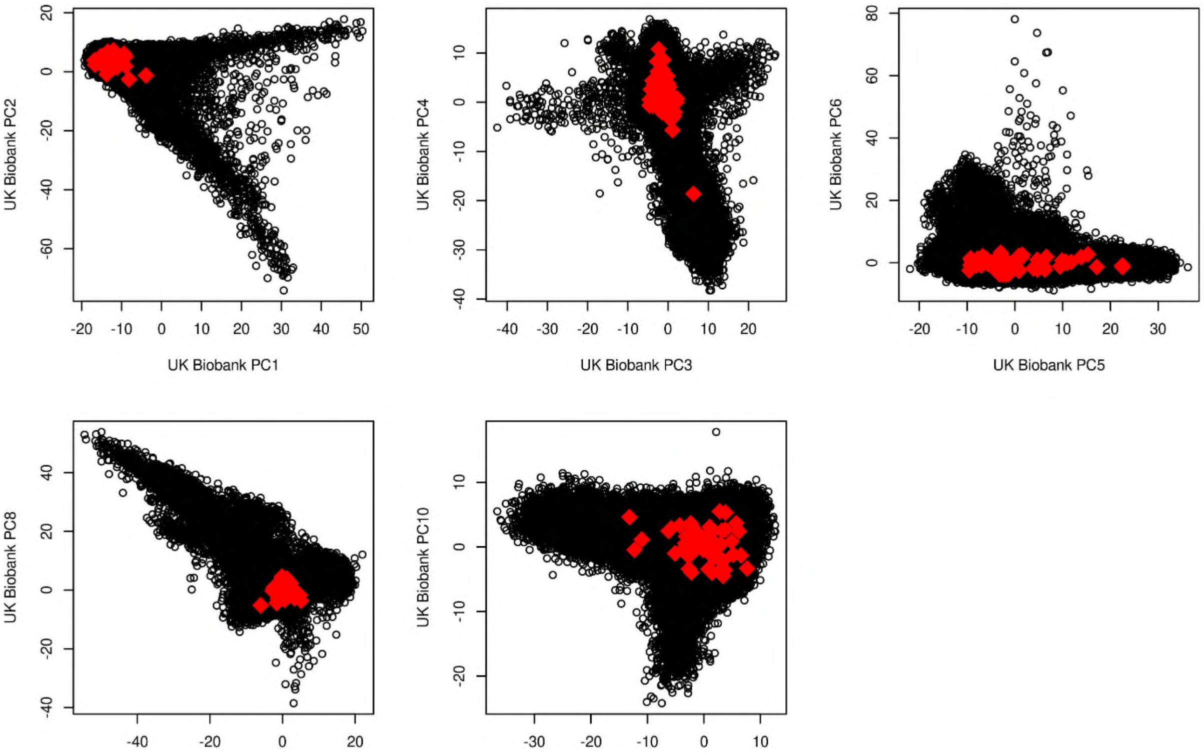

Figure S3 | Geographical distribution of GIGYF1 loss of function carriers by location of birth. GIGYF1 carriers are highlighted in red, all other analysed samples in black. Analysis performed in maximum available sample-size (N=184,972).

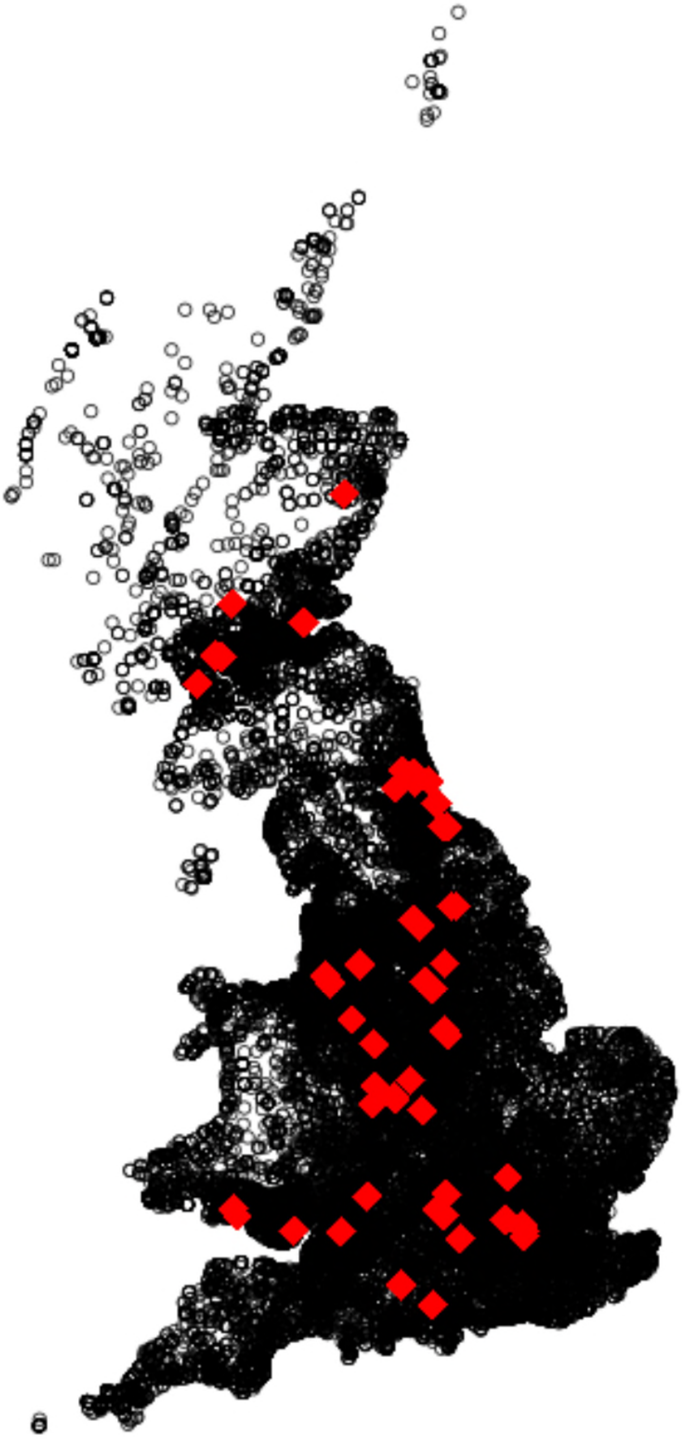

Figure S4 |Regional association of common variants with Type 2 Diabetes, LOY and related traits in the region around GIGYF1 (+/- 500kb). Highlighted variants are the lead variant associated with T2D r2221781 (red) and lead eQTL for *GIGYF1* rs221792 (purple).

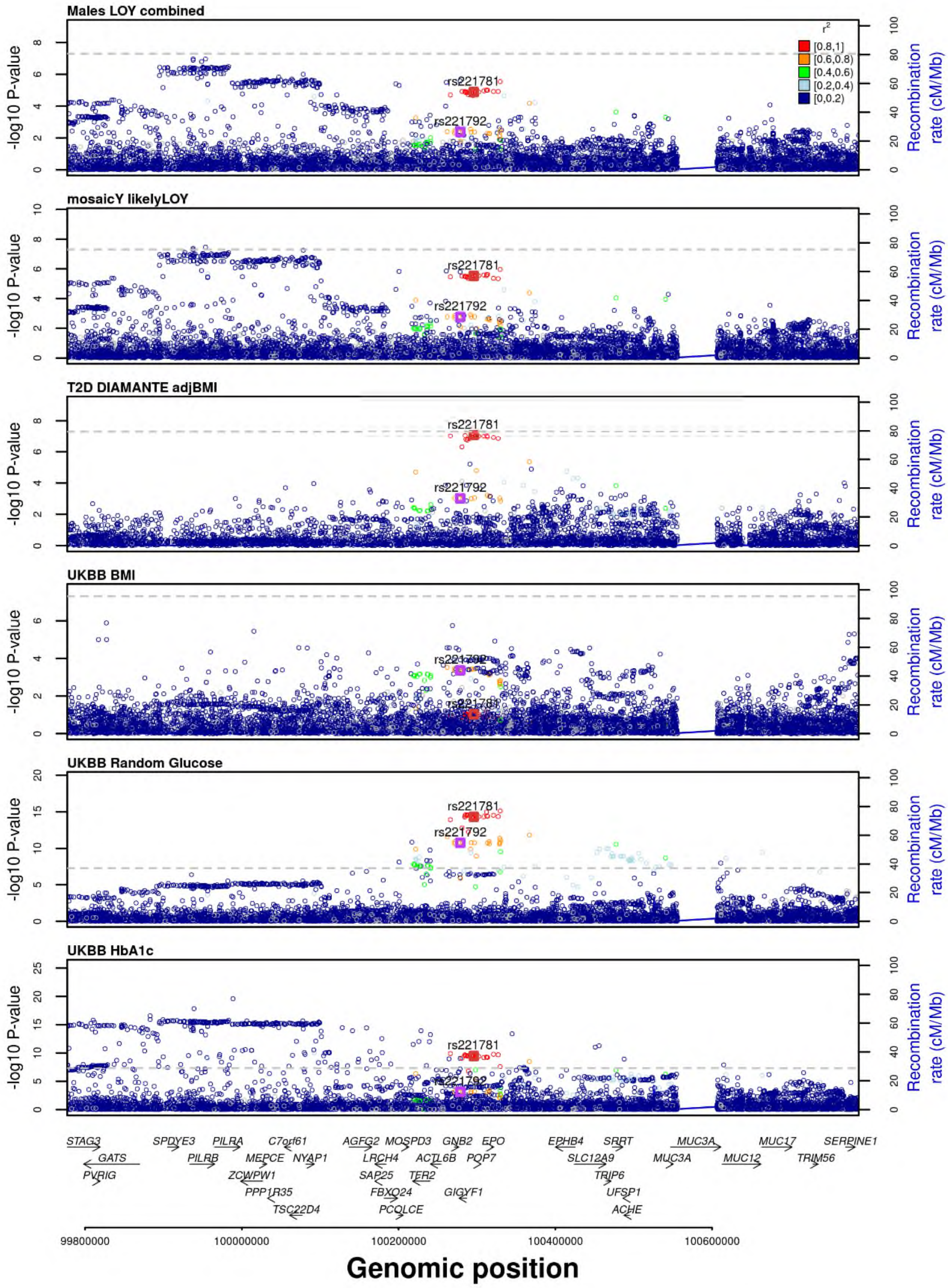

Figure S5 | Multi-tissue eQTL associations in GTEx for common variant rs221781. The solid pink line represents the null effect. Each square represents the beta estimate from a linear regression model of the variant against mRNA transcript abundance. Test statistic is a two-sided P-value and no correction for multiple testing has been made.

Tissue	Samples	NES	p-value	m-value
Skin - Not Sun Exposed (Suprapubic)	517	-0.131	1.0e-9	0.00
Brain - Cerebellar Hemisphere	175	-0.162	5.0e-3	0.973
Liver	208	-0.188	0.006	0.999
Skin - Sun Exposed (Lower leg)	605	-0.203	5.0e-15	1.00
Heart - Left Ventricle	386	-0.219	1.7e-8	1.00
Artery - Tibial	584	-0.220	2.1e-17	1.00
Artery - Coronary	213	-0.221	3.7e-5	1.00
Esophagus - Gastroesophageal Junction	330	-0.238	1.4e-8	1.00
Adipose - Visceral (Omentum)	469	-0.243	7.3e-12	1.00
Esophagus - Mucosa	497	-0.267	7.8e-13	1.00
Spleen	227	-0.268	2.5e-5	1.00
Whole Blood	670	-0.272	7.8e-18	1.00
Testis	322	-0.281	3.4e-11	1.00
Uterus	129	-0.282	8.0e-5	1.00
Breast - Mammary Tissue	396	-0.285	1.4e-15	1.00
Artery - Aorta	387	-0.297	2.0e-17	1.00
Muscle - Skeletal	706	-0.302	3.6e-22	1.00
Nerve - Tibial	532	-0.302	9.2e-24	1.00
Heart - Atrial Appendage	372	-0.303	2.4e-14	1.00
Lung	515	-0.305	2.5e-18	1.00
Brain - Cerebellum	209	-0.305	3.4e-7	1.00
Brain - Anterior cingulate cortex (BA24)	147	-0.315	1.3e-4	1.00
Ovary	167	-0.317	2.0e-6	1.00
Brain - Frontal Cortex (BA9)	175	-0.329	8.0e-7	1.00
Brain - Amygdala	129	-0.334	4.7e-5	1.00
Brain - Cortex	205	-0.335	2.0e-6	1.00
Brain - Hypothalamus	170	-0.335	1.3e-6	1.00
Brain - Hippocampus	165	-0.336	1.6e-7	1.00
Brain - Caudate (basal ganglia)	194	-0.336	1.4e-10	1.00
Adipose - Subcutaneous	581	-0.337	1.4e-32	1.00
Thyroid	574	-0.349	1.5e-27	1.00
Esophagus - Muscularis	465	-0.354	7.3e-27	1.00
Pancreas	305	-0.357	8.6e-10	1.00
Kidney - Cortex	73	-0.357	8.9e-4	1.00
Colon - Transverse	368	-0.361	5.9e-15	1.00
Cells - EBV-transformed lymphocytes	147	-0.365	9.8e-4	1.00
Brain - Nucleus accumbens (basal ganglia)	202	-0.370	5.9e-13	1.00
Brain - Spinal cord (cervical c-1)	126	-0.378	5.3e-6	1.00
Cells - Cultured fibroblasts	483	-0.382	4.3e-32	1.00
Small Intestine - Terminal Ileum	174	-0.387	4.2e-9	1.00
Prostate	221	-0.405	3.4e-9	1.00
Colon - Sigmoid	318	-0.419	1.5e-14	1.00
Brain - Putamen (basal ganglia)	170	-0.431	5.7e-12	1.00
Pituitary	237	-0.436	6.1e-11	1.00
Adrenal Gland	233	-0.438	5.6e-10	1.00
Brain - Substantia nigra	114	-0.449	6.1e-6	1.00
Minor Salivary Gland	144	-0.476	1.8e-9	1.00
Vagina	141	-0.481	5.4e-6	1.00
Stomach	324	-0.499	5.0e-20	1.00

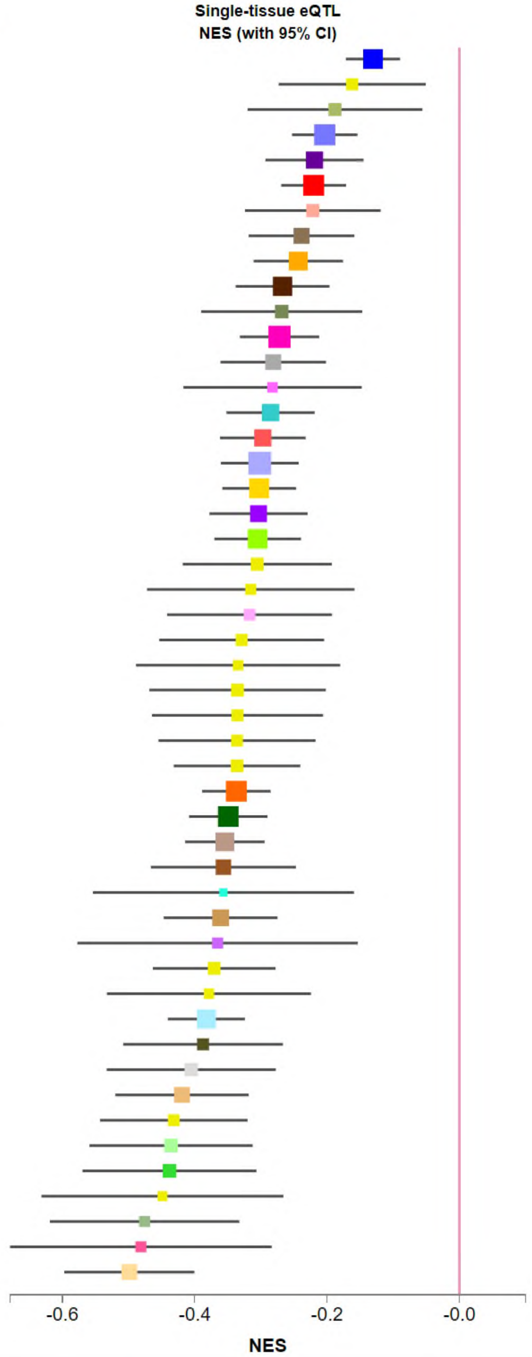

Supplement: Supplementary file 1 — Supplementary Information [file 41467_2021_24504_MOESM1_ESM.pdf]
