# Supplementary material for: GIGYF1 loss of function is associated with clonal mosaicism and adverse metabolic health
Source: Nat Commun. 2021 Jul 7;12:4178. doi: 10.1038/s41467-021-24504-y (PMC8263756; doi:10.1038/s41467-021-24504-y)
Supplement: Supplementary file 2 — Description of Additional Supplementary Files [file 41467_2021_24504_MOESM2_ESM.pdf]

Description of Additional Supplementary Files

File Name: Supplementary Data 1
Description: A comparison of PAR-LOY vs PAR-LOYq association statistics for the previously reported LOY signals

File Name: Supplementary Data 2
Description: : Exome-wide gene-burden association test statistics for LOY

File Name: Supplementary Data 3
Description: Moderate and high impact coding variants identified in GIGYF1

File Name: Supplementary Data 4
Description: Leave-one-out gene burden association analyses for GIGYF1

File Name: Supplementary Data 5
Description: The impact of CADD variant weighting using STAAR for LOY gene burden testing.

File Name: Supplementary Data 6
Description: The association of GIGYF1 loss of function on metabolic traits

File Name: Supplementary Data 7
Description: Phenotypic characteristics of GIGYF1 loss of function carriers

File Name: Supplementary Data 8
Description: Common variant associations on metabolic health at the GIGYF1 locus.
