# Supplementary material for: GIGYF1 loss of function is associated with clonal mosaicism and adverse metabolic health
Source: Nat Commun. 2021 Jul 7;12:4178. doi: 10.1038/s41467-021-24504-y (PMC8263756; doi:10.1038/s41467-021-24504-y)
Supplement: Supplementary file 11 — Reporting Summary [file 41467_2021_24504_MOESM11_ESM.pdf]

nature research

Corresponding author(s): John Perry

Last updated by author(s): Jun 4, 2021

Reporting Summary

Nature Research wishes to improve the reproducibility of the work that we publish. This form provides structure for consistency and transparency in reporting. For further information on Nature Research policies, see our Editorial Policies and the Editorial Policy Checklist.

Statistics

For all statistical analyses, confirm that the following items are present in the figure legend, table legend, main text, or Methods section.

n/a	Confirmed	
☐	☒	The exact sample size (*n*) for each experimental group/condition, given as a discrete number and unit of measurement
☐	☒	A statement on whether measurements were taken from distinct samples or whether the same sample was measured repeatedly
☐	☒	The statistical test(s) used AND whether they are one- or two-sided<br>*Only common tests should be described solely by name; describe more complex techniques in the Methods section.*
☐	☒	A description of all covariates tested
☐	☒	A description of any assumptions or corrections, such as tests of normality and adjustment for multiple comparisons
☐	☒	A full description of the statistical parameters including central tendency (e.g. means) or other basic estimates (e.g. regression coefficient) AND variation (e.g. standard deviation) or associated estimates of uncertainty (e.g. confidence intervals)
☐	☒	For null hypothesis testing, the test statistic (e.g. *F*, *t*, *r*) with confidence intervals, effect sizes, degrees of freedom and *P* value noted<br>*Give P values as exact values whenever suitable.*
☒	☐	For Bayesian analysis, information on the choice of priors and Markov chain Monte Carlo settings
☒	☐	For hierarchical and complex designs, identification of the appropriate level for tests and full reporting of outcomes
☒	☐	Estimates of effect sizes (e.g. Cohen's *d*, Pearson's *r*), indicating how they were calculated

Our web collection on statistics for biologists contains articles on many of the points above.

Software and code

Policy information about availability of computer code

Data collection	No software we used for data collection. Data made available by the UK Biobank study and described previously (Thompson et al, Nature 2019)
Data analysis	All analyses were performed using: BOLT v2.3.4, R (3.3.3) and STAAR (v0.9.5)

For manuscripts utilizing custom algorithms or software that are central to the research but not yet described in published literature, software must be made available to editors and reviewers. We strongly encourage code deposition in a community repository (e.g. GitHub). See the Nature Research guidelines for submitting code & software for further information.

Data

Policy information about availability of data

All manuscripts must include a data availability statement. This statement should provide the following information, where applicable:

- Accession codes, unique identifiers, or web links for publicly available datasets
- A list of figures that have associated raw data
- A description of any restrictions on data availability

All data is available upon application from UK Biobank (www.ukbiobank.ac.uk). Specifically, the exome sequence data resource is described here: https://biobank.ndph.ox.ac.uk/ukb/label.cgi?id=170 and mosaic LOY calls here: https://biobank.ndph.ox.ac.uk/ukb/dset.cgi?id=3094

Field-specific reporting

Please select the one below that is the best fit for your research. If you are not sure, read the appropriate sections before making your selection.

- [x] Life sciences
- [ ] Behavioural & social sciences
- [ ] Ecological, evolutionary & environmental sciences

For a reference copy of the document with all sections, see nature.com/documents/nr-reporting-summary-flat.pdf

Life sciences study design

All studies must disclose on these points even when the disclosure is negative.

Sample size	We used all male UK Biobank participants with exome-sequence data available ('200k release')
Data exclusions	We excluded women from the mosaic Y-chromosome loss analyses and non-european ancestry individuals in all analyses to avoid population stratification effects
Replication	Replication of the rare variant association was not possible and therefore not attempted. We note however that both GIGYF1 and CHEK2 loci have been previously implicated by more common variants.
Randomization	N/A - participants genotype is randomized and fixed at birth and we are assessing the impact of that genotype against phenotype
Blinding	N/A - the statistical tests we are performing do not require blinding.

Reporting for specific materials, systems and methods

We require information from authors about some types of materials, experimental systems and methods used in many studies. Here, indicate whether each material, system or method listed is relevant to your study. If you are not sure if a list item applies to your research, read the appropriate section before selecting a response.

Materials & experimental systems

n/a	Involved in the study	
x		Antibodies
x		Eukaryotic cell lines
x		Palaeontology and archaeology
x		Animals and other organisms
	x	Human research participants
x		Clinical data
x		Dual use research of concern

Methods

n/a	Involved in the study	
x		ChIP-seq
x		Flow cytometry
x		MRI-based neuroimaging

Human research participants

Policy information about studies involving human research participants

Population characteristics	UK Biobank is a national resource that has been described extensively elsewhere (https://www.ukbiobank.ac.uk). Individuals were not directly selected for inclusion in the study on the basis of any disease or health parameter.
Recruitment	UK Biobank: all people aged 40–69 years (men and women) who were registered with the National Health Service and living up to 25 miles from one of the 22 study assessment centers were invited to participate in 2006–2010. Overall, about 9.2 million invitations were mailed to recruit 503,325 participants (a response rate of 5.47%). The individuals in this study are known to be healthier than the general population which may lead to an underestimation of effect sizes (e.g if for example GIGYF1 carriers were too ill to participate in the study)
Ethics oversight	National Research Ethics Service Committee North West–Haydock and all study procedures were performed in accordance with the World Medical Association Declaration of Helsinki ethical principles for medical research.

Note that full information on the approval of the study protocol must also be provided in the manuscript.
